# Intrapersonal Achievement Goals and Underlying Reasons among Long Distance Runners: Their Relation with Race Experience, Self-Talk, and Running Time

**DOI:** 10.5334/pb.280

**Published:** 2016-07-13

**Authors:** Jochen Delrue, Athanasios Mouratidis, Leen Haerens, Gert-Jan De Muynck, Nathalie Aelterman, Maarten Vansteenkiste

**Affiliations:** 1 Ghent University, BE; 2 Hacettepe University, TR

**Keywords:** Self-Determination Theory, Achievement Goal Approach, Intrapersonal Goals, Flow, Self-talk, Basic Need Satisfaction

## Abstract

In a sample of long distance runners, we examined the role of type of intrapersonal achievement goals (i.e., approach versus avoidance) and type of underlying reasons (i.e., autonomous and controlled), assessed prior to the race, as predictors of both pre-race (e.g., race appraisals) and post-race (e.g., flow experience) outcomes. Of 221 (62.4% males) runners, 111 reported pursuing an intrapersonal-approach goal (i.e., doing better than before) as their dominant or preferred achievement goal for the race, while 86 prioritized intrapersonal-avoidance goals (i.e., avoiding to perform worse than before). Regression and path analyses showed that the type of achievement goals predicted none of the outcomes except for running time, with approach goals predicting better performance when compared to avoidance goals. Path analyses revealed that autonomous reasons underlying intrapersonal goal pursuit related positively to pre-race challenge appraisals, performance and, via need satisfaction, to flow experience. Interestingly, controlled reasons positively related to pre-race threat appraisals and positively predicted both positive and negative self-talk, with both yielding opposing relations with flow. These findings complement past research on the intersection between the Achievement Goal Approach and Self-Determination Theory and highlight the value of studying the reasons underlying intrapersonal achievement goals.

Distance running has become a popular recreational sport activity, as illustrated by the increasing participation rates in races like the Marathon of New York and the 20 kilometers of Brussels (Scheerder, Breedveld, & Borgers, 2015). One critical factor to understand runners’ running experience is their motivation for participating in a race and for aspiring certain achievement goals. We relied on Self-determination Theory (SDT: Deci & Ryan, 2000) and the Achievement Goal Approach (AGA: Elliot, 2005) and sought to understand whether the motivational experiences of runners of a popular street race, the “20 km of Brussels”, would relate to their race-appraisals, race experiences, and their actual performance. We focused on runners’ intrapersonal achievement goals (Elliot, Murayama, & Pekrun, 2011), that is, the type of goals that athletes set for themselves in relation to their previous performance, because such goals are highly salient among long distance runners and remain understudied in the sports context.

Specifically, we examined whether runners’ race appraisals, flow and performance would vary as a function of the type of pre-race intrapersonal goal runners set (i.e., approaching success versus avoiding failure) and the reasons for pursuing the goal (i.e., autonomous versus controlled). Further, we considered two different mechanisms, that is, psychological need satisfaction and self-talk, as potential explanatory processes of the hypothesized relation between pre-race goals and underlying reasons on the one hand and flow and performance on the other (see Figure 1). The satisfaction of the psychological needs for autonomy and competence is critical for full task absorption (Kowal & Fortier, 1999), which is conducive to a flow experience. Yet, apart from this more affective mechanism, we also considered the role of self-talk, a more cognitive-oriented process, as it denotes “athletes’ verbalizations to themselves” (Hardy, Hall, & Hardy, 2005). We reasoned that athletes’ self-talk may represent a critical motivational vehicle through which runners’ achievement goals and their underlying reasons may relate to their race experience and racing time.

**Figure 1:**
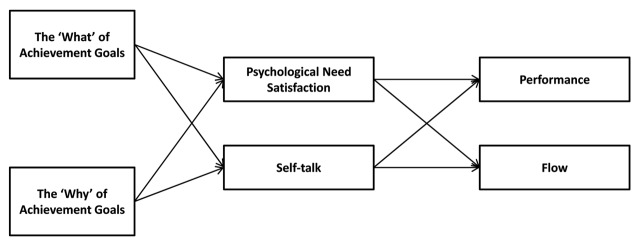
Theoretically Proposed Model.

## Intrapersonal Goals: An Understudied Type of Achievement Goals

Over the past two decades, AGA (Elliot, 2005; Senko, Hulleman, & Harackiewicz, 2011) has been the guiding framework in dozens of studies in the sports literature (e.g., Adie, Duda, & Ntoumanis, 2008; Nicholls, Perry, & Calmeiro, 2014). Goals are “concrete cognitive representations that serve a directional function in motivation by guiding the individual toward or away from a specific possible outcome” (Elliot & Thrash, 2001 p. 143). A goal thus refers to a particular aim or end result one tries to approach or avoid. Within the AGA, six different achievement goals have been discerned depending on how competence and the associated success are defined and valenced.

Three achievement goals have been distinguished as a function of whether competence is defined depending on a) a task-based or absolute standard, b) an intrapersonal or self-based standard, and c) an interpersonal or normative standard (Elliot et al., 2011). With respect to the *valence* dimension, the competence standard can be evaluated positively in which case individuals approach an achievement situation to attain success, or it can be evaluated negatively, in which case individuals are focused on avoiding incompetence or failure (e.g., Elliot et al., 2011). By crossing the dimensions of definition and valence, a taxonomy consisting of six different achievement goals is obtained. To illustrate, athletes adopt a task-approach goal when they are focused on mastering the requirements of the task and a task-avoidance goal when they want to avoid falling short of such requirements. Instead, when athletes aim to do better or avoiding doing worse compared to their former performances on a similar task, they are said to hold, respectively, an intrapersonal-approach and an intrapersonal-avoidance goal. Finally, when athletes aim to do well in comparison with others or aim to avoid performing worse than others, they are said to endorse a normative-approach and a normative-avoidance goal, respectively.

The number of achievement goals scholars have studied has varied depending on their research questions, interests, and preferences. In sports contexts, most research has examined the correlates of task-based and normative goals. In general, a host of primarily cross-sectional studies have shown that task-approach goals are positively related to challenge appraisals (e.g., Adie et al., 2008; Nicholls et al., 2014), positive self-talk (Zourbanos, Pappaioannou, Argyropolou, & Hatzigeorgiadis, 2014), enjoyment (e.g., Morris & Kavussanu, 2009), and performance (e.g., Van Yperen, Blaga, & Postmes, 2014), while being negatively related to threat appraisals (e.g., Adie et al., 2008), negative self-talk (Zourbanos et al., 2014), cognitive anxiety (Morris & Kavussanu, 2009), and self-handicapping (e.g., Chen, Wu, Kee, Lin, & Shui, 2009). Task-avoidance goals, on the contrary, were found to relate positively to threat appraisals (Adie et al., 2008; Nicholls et al., 2014), cognitive anxiety (Morris & Kavussanu, 2009), and self-handicapping (Chen et al., 2009). Similar to task-approach goals, normative-approach goals have been found to relate positively to performance (Van Yperen et al., 2014), challenge (Adie et al., 2008), as well as threat appraisals (Adie et al., 2008) and negatively to self-handicapping (Chen et al., 2009). Contrary to their approach oriented counterparts, normative-avoidance goals are positively related to self-handicapping (e.g., Chen et al., 2009), cognitive anxiety (Morris & Kavussanu, 2009) and threat appraisals, but negatively to challenge appraisals (Adie et al., 2008).

To date, intrapersonal goals have received far less attention within the AGA framework, presumably because they were only fairly recently conceptually and empirically differentiated from task-based goals (Elliot et al., 2011). This is unfortunate because intrapersonal goals may be heavily prominent among athletes and carry a high ecological validity. Indeed, for most athletes, improving their skills or performance is a primary goal and a key factor that influences their motivational functioning (see Martin, 2006; Harwood, Hardy, & Swain, 2000). To fill this void, we investigate herein whether athletes participating in a long distance running race would favor intrapersonal over normative goals. In addition, we examined whether the type of *dominant* or *preferred* achievement goal (Van Yperen, 2006) of runners would relate to pre-race appraisals and expected running time, and during the race itself to self-talk, need satisfaction, flow experience, and finally actual running performance.

A few previous studies have examined the correlates of intrapersonal goals outside the sports domain. In an initial examination, Van Yperen (2006) found that two thirds of the learners favored intrapersonal above normative achievement goals, with intrapersonal-approach goals being the most frequently selected. Further, learners with a dominant intrapersonal-avoidance goal scored lowest on intrinsic motivation and self-efficacy compared to most of the other achievement goal profiles. In a subsequent experimental study, Van Yperen, Elliot and Anseel (2009) showed that the activation of an intrapersonal-avoidance goal, relative to an intrapersonal-approach goal, results in lower levels of performance improvement. More recently, Elliot et al. (2011) reported that, when controlling for the shared variance between all six identified achievement goals, intrapersonal-approach and intrapersonal-avoidance goals yielded, respectively, a unique positive and a unique negative relation to learners’ energy, but both were unrelated to intrinsic motivation.

Although not directly grounded in the AGA, Martin’s (2006) work on personal best goals, which he defined as “personalized goals or standards of excellence that match or exceed one’s previous best” (Martin & Liem, 2010; p. 264), is worth being mentioned because of its resemblance with intrapersonal-approach goals. Similar to intrapersonal approach goals, personal best goals appear quite adaptive as they relate positively to enjoyment, class participation, persistence, and achievement among learners.

Overall then, intrapersonal goals have received far less attention within the AGA and were almost exclusively studied among learners (but see Van Yperen, Hamstra, & Van Der Klauw, 2011). This leaves the question unanswered whether these goals, relative to normative goals, would yield different affective and cognitive outcomes among athletes. Specifically, flow and actual performance constitute the critical outcomes in the current study. Flow refers to an optimal psychological state in which a person is totally immersed in an activity and has positive experiences like freedom of self-consciousness and enjoyment of the process (Jackson & Marsh, 1996). With regard to performance, running time constitutes an important outcome in a running race. Besides, we were also interested whether the intrapersonal goals would relate to how runners appraise the upcoming race (i.e., as a challenge or a threat).

## Not All Intra-Personal Goals Are Equally Motivated: Examining their Underlying Reasons

Apart from the gradual extension of the number of studied achievement goals, another important evolution within the field concerns the revision of the achievement goal concept as such (Vansteenkiste, Lens, Elliot, Soenens, & Mouratidis, 2014). Specifically, Elliot and Thrash (2001) maintained that achievement goals should be exclusively defined based on the type of pursued standard, whereas all other aspects, including feelings, reasons, and attributions, should be removed from the achievement goals definition as they represent peripheral rather than central features. This reconceptualization represented an important departure from the initial conceptualization of achievement goals (Nicholls, 1984; Dweck, 1986) according to which achievement goals had been conceptually interwoven with specific underlying reasons. For instance, the pursuit of normative goals was originally conceived as ego-involved as performance-oriented individuals were supposed to outperform their peers to prove, or boost, their self-worth and value. The separation of the reasons (i.e., “why” of achievement goals) from the type of pursued aims (i.e., “what” of achievement goals) created the possibility to systematically investigate the role of different types of reasons underlying achievement goals (Lens & Vansteenkiste, 2006; Vansteenkiste, Lens et al., 2014). To illustrate, athletes may no longer solely pursue normative goals out of ego-concerns but also to meet external pressures or because they may consider competing with others as an exciting opportunity and challenge (Reeve & Deci, 1996; Vansteenkiste, Mouratidis, & Lens, 2010).

Vansteenkiste, Smeets and colleagues (2010) argued that one framework that is ideally suited to study a diversity of reasons that may drive individuals’ achievement goal pursuit is Self-Determination Theory (SDT; Deci & Ryan, 2000). Specifically, athletes can pursue goals because they find them enjoyable, challenging, or personally significant (i.e., autonomous reasons) or because they feel internally or externally pressured to do so (i.e., controlled reasons). An increasing number of mostly cross-sectional studies in diverse domains, including education (e.g., Gaudreau, 2012) and work (e.g., Gillet, Lafrenière, Vallerand, Huart, & Fouquereau, 2014), have examined the unique and interactive contribution of achievement goals and underlying reasons in the prediction of outcomes, and found these reasons to account for substantial and unique variance above and beyond the achievement goals themselves (see Vansteenkiste, Lens et al., 2014 for an overview).

To the best of our knowledge, only three such studies were conducted in the sports context, albeit mostly in team sports. Focusing on the reasons underlying normative goals among amateur soccer players, Vansteenkiste, Mouratidis et al. (2010) found controlled reasons to relate positively to immoral functioning (i.e., aggressive play), whereas autonomous reasons were positively associated with positive emotional outcomes. Next, in a study among volleyball players, who were followed during multiple consecutive competitive games (Vansteenkiste, Mouratidis, Van Riet, & Lens, 2014), game-to-game variation in the autonomous regulation of task-approach goals related positively to game-to-game variation in affective (e.g., enjoyment, performance satisfaction) and behavioral outcomes (e.g., prosocial behavior). Finally, Gaudreau and Braaten (this issue) reported that autonomous reasons underlying both task-approach and normative-approach goals related positively to positive affect and subjective performance among athletes from various sports, whereas controlled reasons were related to less positive and more negative affect. Moreover, reasons and achievement goals interacted such that autonomous reasons amplified the positive association between task-approach goals and desirable outcomes.

Theoretically, the reason why autonomous regulation yields various benefits is because it allows for greater satisfaction of the psychological needs for autonomy (i.e., experiencing a sense of volition), competence (i.e., feeling effective), and relatedness (i.e., experiencing closeness) (Deci & Ryan, 2000). In contrast, a controlled regulation may engender experiences of need frustration (e.g., Haerens, Aelterman, Vansteenkiste, Soenens, & Van Petegem, 2015). Consistent with this argument, Gillet et al. (2014) found psychological need satisfaction to explain the positive contribution of autonomous reasons underlying normative-approach goals to affective outcomes in the goal process.

## Present Research

The present study aimed to extend the limited body of work on the “what” and “why” of achievement goals by (a) focusing on an underexamined achievement goal (i.e., intrapersonal goals), (b) sampling athletes participating in an individual instead of a team sport (i.e., runners), (c) adopting a prospective instead of cross-sectional research design in the prediction of outcomes that are highly appreciated in competitive sports such as flow and performance, (d) including an objective rather than a self-reported performance indicator, and (e) considering the role of both a more affective (i.e., need satisfaction) and a cognitive (i.e., self-talk) explanatory mechanism.

Specifically, we adopted a prospective design, thereby including a host of pre- and post-race variables. The inclusion of both pre- and post-race variables allowed us to examine whether the type of pursued achievement goal (the “what”) and its underlying reasons (the “why”) would not only relate to how runners appraise the race (i.e., as a challenge or a threat) and what time they set as a target, but also whether these motivational dynamics would carry over into how they eventually come to experience the race and how well they actually perform (Figure 1).

We pursued the following five hypotheses. First, we investigated the prevalence of different types of personal achievement goals among long distance runners. As the participating runners are experienced amateurs, with many of them having a fairly clear view of their personal best time, we hypothesized that most of them would select an intrapersonal goal as their primary or dominant goal for the race (Van Yperen, 2006).

Second, we explored whether runners would display a different pattern of outcomes depending on their selected dominant achievement goal. Because approach goals orient runners to the possibility of success, we expected runners with a dominant approach goal, either intrapersonal or normative, to perceive the race more as a challenge, to aspire a sharper time, to experience greater flow and psychological need satisfaction during the race and to run faster compared to runners adopting an avoidance goal.

Third, concerning the ‘why’ of achievement goals we expected that autonomous and controlled reasons underlying intrapersonal goals would explain additional variance in the outcomes above and beyond the variance explained by the ‘what” of achievement goals. Specifically, we hypothesized autonomous reasons to relate to a positive pattern of outcomes involving greater challenge appraisal, need satisfaction, and flow experience as well as better performance. In contrast, controlled reasons would relate to a more negative pattern of outcomes involving greater threat appraisals, more negative self-talk, less need satisfaction, and less flow.

Fourth, we examined whether the ‘what’ and ‘why’ of intrapersonal goals would interact in the prediction of outcomes. While Gaudreau (2012) reported fairly systematic evidence for such interactions in the case of both task-approach and normative-approach goals, other studies provided only partial (Benita, Roth, & Deci, 2014; Gaudreau & Braaten, this issue; Gillet et al., 2014) or no evidence at all for such interactions (Vansteenkiste et al., 2010). It is possible though that the reasons underlying intrapersonal goals alter the perceived meaning of the achievement goals themselves, such that the effects of goal-contents vary as a function of these reasons. Alternatively, reasons may exacerbate the hypothesized effects of particular goal-contents, such that, for instance, particular goal-contents (e.g., avoidance goals) may in combination with particular reasons (e.g., controlled) yield a surplus effect not accounted for by the two main effects.

Fifth, as depicted in our theoretical Figure 1, we explored whether runners’ self-talk and experienced need satisfaction during the race can help to explain the effects of the ‘what’ and ‘why’ of achievement goals on flow experience and performance. Whereas need satisfaction, as a more affective process, has received considerable prior attention in the SDT-literature (e.g., Chen, Vansteenkiste, Beyers, Soenens, & Van Petegem, 2013), self-talk, as a more cognitive mechanism, has not been explored. We reasoned that self-talk represents a mental tool (Schüler & Langens, 2007; Blanchfield, Hardy, Morree, Staiano, & Marcora, 2014) to regulate ongoing behavior and affective experiences in a goal striving context, thereby allowing one to either boost or undermine experiences of flow and performance. Self-talk has been found to promote greater attention and performance (e.g., Hatzigeorgiadis, Zourbanos, Mpoumpaki, & Theodorakis, 2009; Van Raalte et al., 1995), to help marathon runners counter a “psychological crisis” during the race (Schüler & Langens, 2007), and can be predicted by one’s pursued achievement goals (e.g., Zourbanos et al., 2014). We hypothesized that the pursuit of intrapersonal-avoidance goals, relative to intrapersonal-approach goals, and the controlled regulation of the goals would go together with more negative self-talk due to the pressure and anxiety associated with avoidance goals and its controlled regulation (see Oliver, Hardy, & Petherick, 2008). We were more ambivalent with respect to the effects of autonomous reasons, as autonomously motivated runners are more likely to get fully immersed in the race (Kowal & Fortier 1999), thereby leading them to experience greater need satisfaction and flow without necessarily prompting them to engage in any self-talk at all. On the other hand, to the extent they are engaged in self-talk, such self-talk may be rather positive, which in turn would associate with more flow experience.

## Method

### Participants and Procedures

We recruited participants through two different channels. First, we contacted two Flemish non-governmental organizations (NGO), which encourage their members to take part in the 20 kilometers of Brussels, to participate in the present study. To promote the study amongst the members of these two organizations, a flyer was composed with basic information regarding the purpose of the study and a link to an online questionnaire. This flyer was distributed to individuals who had subscribed for the race through the NGO one week before the race. Second, during the week before the race, the study was promoted on the social medium of the race organization. As such, participants were able to get access to the online questionnaire. All the participants filled in the pre-race questionnaire between one and six days before the race. During this first assessment, 246 (63.4% males) participants (236 Belgians, 4 Dutch, 1 Belgian-Portuguese, 1 Italian, Portugese, Polish, Spanish, and Jamaican) were asked to provide their e-mail address through which we invited them to fill in the post-race assessment. One day following the street race, all participants got an inviting e-mail, of whom 180 completed the post-race assessment (81.4% retention), at the latest seven days after the race. Only one participant completed the post-race questionnaire nine days later.

### Measures

#### Pre-race assessment

***Dominant Achievement Goal***. Runners’ dominant or preferred achievement goal (Van Yperen, 2006) was assessed via a rank order method (see Vansteenkiste, Mouratidis et al., 2014). Having read the stem “In the upcoming race I find it most important…”, the participants were asked to rank order the following four achievement goals: “… to do better than others” (normative-approach goal), “… not to do worse than others” (normative-avoidance goal), “… to do better than before” (intrapersonal-approach goal) and “… not to do worse than before” (intrapersonal-avoidance goal). The goal that was ranked first was considered the runners’ dominant achievement goal.

***Reasons Underlying the Dominant Achievement Goal***. Once runners had identified their dominant achievement goal for the upcoming race, they were given a set of items that tapped into the autonomous and controlled reasons for pursuing their self-identified dominant achievement goal (Vansteenkiste, Mouratidis et al., 2014). After the stem “For the upcoming race I aim to pursue the goal I have ranked first because…”, there were sixteen items purporting to probe four different regulations, namely external regulation (e.g., “… others would appreciate me”; α = .80), introjected regulation (e.g., “… I would feel guilty if I would not”; α = .72), identified regulation (e.g., “… I totally agree with this goal”; α = .71), and intrinsic motivation (e.g., “… I find it a challenge to aim for this goal”; α = .75). A five-point Likert-type scale was used anchored from 1 (*I totally disagree*) to 5 (*I totally agree*). Scores for controlled and autonomous reasons were computed by averaging, respectively the external and introjected regulation items (α = .84) and identified and intrinsic regulation items (α = .82). The creation of these two composite scores was also empirically justified as a principal component analysis provided evidence for the extraction of two distinct factors representing autonomous (λ = 3.14) and controlled motives (λ = 4.62), which explained, respectively, 19.61% and 28.85% of the total variance.

***Race Appraisals***. Runners’ race appraisals were assessed via the *Challenge and Threat Construal Questionnaire* (McGregor & Elliot, 2002), which was translated and adapted for the purposes of the current study. This instrument consisted of five items probing the perception of challenge (e.g., “I view the upcoming race as a challenge”; α = .65) and of five items asking for the perception of threat (e.g., “I am dreading the upcoming race”, α = .77). Participants answered on a seven-point Likert-type scale with answers ranging from 1 (*Not at all true of me*) to 7 (*Completely true of me*).

#### Post-race assessment

***Self-talk***. Runners’ self-talk during the race was assessed via a translated version of the *Automatic Self-Talk Questionnaire for Sports* (ASTQ-S; Zourbanos, Hatzigeorgiadis, Chroni, Theodorakis, & Papaioannou, 2009). This self-talk instrument includes a positive (α = .92) and negative self-talk (α = .88) factor, each consisting of four subscales. The four positive subscales were *psyching up* (5 items; e.g., “Do your best”), *anxiety control* (4 items; e.g., “Don’t get upset”), *confidence* (5 items; e.g., “I feel strong”), and *instruction* (5 items; e.g., “Concentrate”). The four negative scales were *worry* (7 items; e.g., “I am not going to make it”), *disengagement* (5 items; e.g., “I want to stop”), *somatic fatigue* (5 items; e.g., “I am tired”), and *irrelevant thoughts* (4 items; e.g., “what will I do later tonight?”). The five-point Likert scale was answered from 0 (*never*) to 4 (*very often*) to indicate how often runners had such thoughts during the race. A second-order principal component analysis, including the various subscales instead of items, with promax rotation indicated that two factors could best be retained. All four positive subscales loaded on the first factor (λ = 8.50; explained variance 21.79%), while all negative subscales, but irrelevant thoughts, loaded on the negative factor (λ = 5.99; explained variance 15.37%). Although irrelevant thoughts did not load on any factor, we retained this subcomponent in the computation of the composite score of negative self-talk in light of prior empirical findings and on theoretical grounds.

***Psychological Need Satisfaction***. An adapted version of the *Basic Need Satisfaction in Sport Scale* (BNSSS; Ng, Lonsdale & Hodge, 2011) was used to assess runners’ autonomy and competence need satisfaction. After the stem “During the race I felt…”, there were four items gauging competence need satisfaction (e.g., “I could handle this challenge”; α = .80), and six items measuring autonomy, (e.g., “I was doing what I wanted to do” and “I was participating willingly”; α = .79) All the answers were provided on the seven point Likert type format from 1 (*Not at all true of me*) to 7 (*Completely true of me*).

***Flow***. Runners were asked for their flow experience via a translated and adapted version of the *Flow State Scale* (FSS; Jackson & Marsh, 1996). As the balance between challenges and skills is considered a precondition rather than a central part of flow (Kawabata & Mallet, 2011), we left out this subscale. The runners indicated to what extent during the race they concentrated on their race (e.g., “My attention was focused entirely on what I was doing; α = .77); felt that their actions were merging with their self (e.g., “things just seemed to happen automatically; α = .67); lost self-consciousness (e.g., “I was not concerned with how I was presenting myself” ; α = .68); had sense of control, without actively trying to exert control (e.g., “I felt like I could control what I was doing”; α = .83); experienced transformation of time (e.g., “The way time passed seemed to be different from normal”; α = .74); and had autotelic experiences (e.g., “I found the experience extremely rewarding”; α = .88). All the answers for the six four-item subscales were provided on a seven-point Likert type format ranging from 1 (*I totally disagree*) to 7 (*I totally agree*). An average score from the six subscales was computed and used as an index of athletes’ flow experiences (α = .84).

## Results

### Preliminary analyses

After inspection of the data, several missing values in certain outcomes were found. For instance, among the 246 athletes who completed the pre-race assessment 24 (9.8%) failed to finish the 20 km run and, as a result, we had no information regarding their performance. Likewise, we had 22 (10.2%) missing values for the dominant goal, 35 (14.2%) missing values for challenge and threat, 62 (25.2%) for positive and negative self-talk, and 70 (28.5%) for flow experience. Although a missing data test with expectation maximization algorithm was statistically nonsignificant (Little’s MCARC test χ^2^[(57] = 58.06, *p* = 44, *ns*.) suggesting that missing values were most likely missing at random, we opted for listwise deletion for each set of analyses that we performed.

Independent sample *t-*tests with the available data indicated that males appraised the race as less threatening (*M* = 1.93; *SD* = 0.91; *t*(209) = –2.88, *p* < .01), were more ambitious (*M* = 105.19; *SD* = 16.61; *t*(150.877) = –7.39, *p* < .001), and ran faster (*M* = 106.30; *SD* = 23.11; *t*(208) = –7.36, *p* < .001) compared to females (*M* = 2.31; *SD* = 0.96; *M* = 124.55; *SD* = 20.39; *M* = 129.74; *SD* = 19.12). Therefore we decided to control for gender in all regressions and path-analyses. Further, age related negatively to controlled reasons (*r* = –.24, *p* < .01), challenge (*r* = –.28, *p* < .01) and threat appraisal (*r* = –.34, *p* < .01) before the race and to negative self-talk (*r* = –.19, *p* < .001) during the race. By consequence we controlled for age as well. The bivariate correlations among the measured constructs are reported in Table 1.

**Table 1:** Bivariate Correlations of the Measured Variables of the Study among All Participants.

Variables	1	2	3	4	5	6	7	8	9	10	11	12	13

*Background variables*													
1. Gender	–												
2. Age	–.13*	–											
*Pre-race measures*													
3. Autonomous reasons	.01	–.08	–										
4. Controlled reasons	.01	–.24**	.25**	–									
5. Challenge appraisal	.11	–.28**	.45**	.25**	–								
6. Threat appraisal	.20**	–.34**	.06	.43**	.10	–							
7. Aspired performance	.48**	.11	–.22**	–.14*	.07	.12	–						
*Post-race measures*													
8. Positive self-talk	–.13	.01	.19**	.30**	.26**	.16*	.01	–					
9. Negative self-talk	.04	–.19*	.11	.30**	.01	.29**	.04	.23**	–				
10. Autonomy satisfaction	–.05	.07	.39**	–.06	.28**	–.24**	.08	.18*	–.25**	–			
11. Competence satisfaction	–.11	–.07	.33**	.14	.39**	–.14	–.25**	.28**	–.22**	.49**	–		
12. Flow	–.04	–.01	.18*	.08	.28**	–.05	.02	.26**	–.34**	.34**	.44**	–	
13. Actual performance	.47**	.11	–.20**	–.07	.03	.14	.91**	.03	.14	.09	–.25**	.00	–

*Note*. *p < 05. **p < .01.

### Hypothesis 1: Prevalence of Dominant Achievement Goals

Only few athletes ranked as most important either normative-approach (*N* = 11; 5.0%) or normative-avoidance goals (*N* = 13; 5.9%). The large majority of them reported either intrapersonal-approach (*N* = 111; 50, 2%) or intra-personal avoidance goals (*N* = 86; 38.9%) as their dominant goal. A chi-square test examining the distribution of the dominant goal frequencies was significant, χ*²*(3) = 141.12, *p* < .01. As such, the participants were not equally distributed over the goals. In particular, the respective odds to report intrapersonal-approach goals over normative-approach and normative-avoidance goals were, respectively, 19.3 and 16.1 times higher. Likewise, the odds for an athlete to select intrapersonal-avoidance goals over the normative-approach and normative-avoidance goals were, respectively, 12.2 and 10.2 times higher. Finally, as for the intrapersonal goals themselves, the odds for an athlete to report intrapersonal-approach goal over intrapersonal-avoidance goal as a dominant goal was 1.58 times higher. Taken together, these results suggest that intrapersonal-approach goals were most salient, followed by intrapersonal-avoidance goals and normative goals.

### Hypothesis 2: Differences between Dominant Goal Profiles

Next, we examined whether the athletes differed in any of the pre-race or post-race outcomes as a function of their dominant goal endorsement. To avoid extensive listwise deletion due to missing cases in athletes’ post-race self-reports, we performed two sets of multivariate analysis of variance (MANOVA), one involving the pre-race measures (i.e., challenge, threat, autonomous and controlled reasons underlying dominant achievement goal and athlete’s aspired time) and one containing the post-race measures (i.e., positive and negative self-talk, need satisfaction, flow, and actual performance). Both sets of dependent variables were analyzed as a function of the dimensions of competence definition (i.e., intrapersonal vs. normative) and valence (i.e., approach vs. avoidance) and their interaction. Regarding the pre-race assessment variables, there was a main effect for competence valence, Wilk’s Λ = .941, *F*(5, 198) = 2.48, *p* < .05, partial η^2^ = .06, but not for competence definition, Wilk’s Λ = .955, *F*(5, 198) = 1.86, *p* = .10, nor for the competence valence by definition interaction, Wilk’s Λ = .979, *F*(5, 198) = 0.83, *p* = .53. The follow-up ANOVAs for the competence valence dimension (controlling for inflated type I errors according to the Bonferroni procedure) showed statistically significant differences in aspired performance time only (*F*[1, 202] = 10.29, *p* < .01, partial η^2^ = .05). In particular, athletes who endorsed an approach goal aspired to run faster (*M* = 105.83 minutes; *SD* = 2.97) when compared with their counterparts who endorsed an avoidance goal (*M* = 119.41 minutes; *SD* = 3.02).

Regarding the post-race assessment variables, there was, again, a main effect for the valence dimension (i.e., approach versus avoidance), Wilk’s Λ = .898, *F*(7, 157) = 2.57, *p* < .05, partial η^2^ = .10, but not for the competence definition, Wilk’s Λ = .954, *F*(7, 157) = 1.07, *p* = .39, nor for the definition by valence interaction, Wilk’s Λ = .957, *F*(7, 157) = 1.00, *p* = .43. The follow-up ANOVAs for the valence dimension (after Bonferroni adjustment for inflated type I errors) showed marginally significant differences in negative self-talk (*F*[1, 163] = 6.74, *p* = .01, partial η^2^ = .04) and actual performance (*F*[1, 163] = 5.30, *p* = .02, partial η^2^ = .03). Inspection of the means revealed that athletes who favored a dominant approach goal reported less negative self-talk (*M* = 0.58; *SD* = 0.09) and performed better (*M* = 113.03 minutes; *SD* = 3.83) than athletes with a dominant avoidance goal (*M* = 0.88; *SD* = 0.07 and *M* = 124.63 minutes; *SD* = 3.22). The means and standard deviations as a function of dominant goal endorsement are shown in Table 2.

**Table 2:** Mean-group Comparisons between Runners as a Function of Chosen Dominant Achievement Goal.

	Dominant or Preferred Goal									
Variables	Intra-approach goals (*N* = 111; 63.1% males)		Intra-avoidance goals (*N* = 86; 59.3% males)		Normative-approach goals (*N* = 11; 81.8% males)		Normative-avoidance goals (*N* = 13; 61.5% males)		*F*-statistic	

*Pre-race assessment*										
1. Challenge appraisal	5.19	(0.85)	4.93	(0.80)	5.02	(1.15)	5.22	(1.11)	(3, 207)	1.43
2. Threat appraisal	2.00	(0.84)	2.02	(0.95)	2.22	(1.04)	2.91	(1.39)	(3, 207)	3.37
3. Aspired time	106.67	(17.33)	119.33	(21.50)	105.33	(20.50)	121.83	(15.67)	(3, 211)	8.47*
*Post-race assessment*										
4. Positive self-talk	1.89	(0.69)	1.76	(0.69)	2.17	(0.48)	2.20	(0.51)	(3, 176)	2.03
5. Negative self-talk	0.67	(0.46)	0.68	(0.40)	0.51	(0.20)	1.06	(0.72)	(3, 176)	3.01
6. Autonomy satisfaction	6.36	(0.71)	6.27	(0.74)	6.62	(0.45)	6.41	(0.76)	(3, 173)	0.62
7. Competence satisfaction	5.49	(0.98)	5.17	(0.93)	5.96	(0.55)	5.36	(1.00)	(3, 173)	2.34
8. Flow	3.42	(0.49)	3.47	(0.44)	3.38	(0.35)	3.47	(0.41)	(3, 168)	0.19
9. Actual performance	109.58	(18.50)	123.42	(23.05)	109.45	(23.23)	125.32	(15.20)	(3, 201)	8.08*

*Note*. *p ≤ .0038. Due to Multiple Comparisons, alpha was set at the .0038 level.

Because the reasons underlying the dominant achievement goal were anchored with the self-selected dominant goal and because only a minority of the runners endorsed normative goals, we were forced to drop the normative-oriented athletes when addressing the role of the reasons underlying achievement goals (as was also the case in Vansteenkiste, Mouratidis, et al., 2014).

### Hypothesis 3 and 4: Contribution of the “what” and “why” of intrapersonal goals

Focusing on athletes adopting a dominant intrapersonal goal (*N* = 197), we examined to what extent goal content (i.e., approach vs. avoidance), the type of reasons underlying its endorsement, and the two-way interactions between goal content and reasons predicted pre-race and post-race outcomes by means of hierarchical regression analyses (see Table 3). The background characteristics of age and gender, along with the goal type (intrapersonal-approach vs. intrapersonal-avoidance) were entered in Step 1, the autonomous and controlled underlying reasons were added in Step 2, while in Step 3 all the two-way interactions were considered. Step 3 is not addressed in Table 3, because only one two-way interaction reached significance.

**Table 3:** Hierarchical Regression Analyses for Pre-race and Post-race Measured Variables.

	Pre–race outcomes			Post-race outcomes					
	
Predictors	Challenge (*N* = 188)	Threat (*N* = 188)	Aspired time (*N* = 184)	Positive self–talk (N = 159)	Negative self–talk (*N* = 159)	Autonomy satisfaction (*N* = 158)	Competence satisfaction (*N* = 158)	Flow (*N* = 153)	Performance (*N* = 176)

Step 1									
Gender	.08	.18**	.52**	–.14	.02	–.05	–.13	–.06	.51**
Age	–.27**	–.32**	.14*	.01	–.18*	.01	–.10	–.01	.16*
Intrapersonal goals	.11	–.06	–.28**	.10	–.04	.06	.14†	–.05	–.27**
*F*	7.43**	10.62**	34.07**	1.60	1.77	0.32	2.55	0.31	32.11**
Adjusted *R*^2^	.09	.13	.35	.01	.01	.00	.03	.00	.34
Step 2									
Gender	.08	.18**	.52**	–.15^05^	.01	–.04	–.13	–.06	.51**
Age	–.23**	–.25**	.12^05^	.07	–.14	.01	–.07	.01	.16*
Intrapersonal goals	.02	–.09	–.24**	.04	–.07	–.02	.07	–.10	–.24**
Autonomous reasons	.41**	–.06	–.16**	.12	.02	.47**	.34**	.22*	–.15*
Controlled reasons	.10	.38**	–.04	.28**	.24**	–.17*	.02	.03	.04
F change in *R*^2^	23.97**	16.46**	4.27*	9.32**	4.69*	19.26**	10.23**	3.86**	2.94^06^
Adjusted *R*^2^	.27	.26	.37	.11	.06	.18	.13	.02	.36

*Note*. Intrapersonal goals: 0 = avoidance goal; 1 = approach goal. *p < .05. **p < .01

**Figure 2:**
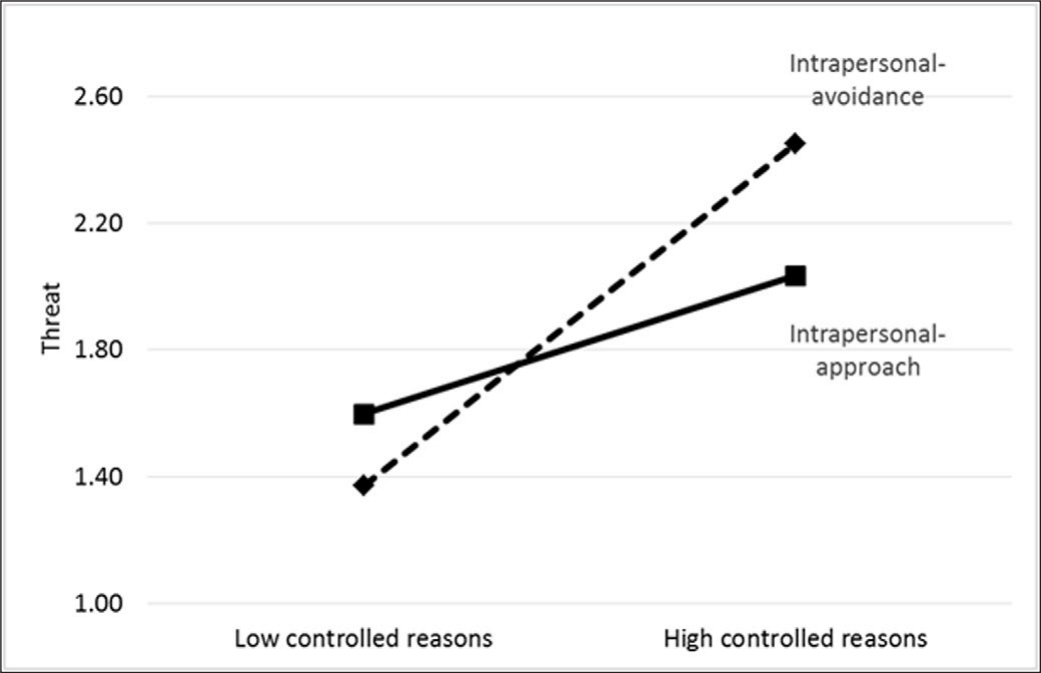
The Interaction between Intrapersonal Goals (Approach vs. Avoidance) and Controlled Reasons Underlying Their Pursuit.

Concerning the pre-race measures, and in partial support of our hypothesis, intrapersonal-approach goals were positive predictors of challenge and aspired time. When reasons were also added in the model, autonomous reasons emerged as a positive predictor of both challenge and aspired time. Controlled reasons emerged as positive predictor of both challenge and threat appraisals. In addition, a statistically significant interaction between goal-content and controlled reasons was found for threat appraisals (*F* change [2, 182] = 3.87, change in adjusted *R*^2^ = .03, *β* = –.26, *p* < .01). The interaction is shown in Figure 2. A test of simple slope indicated that controlled reasons yielded a particular strong relationship with threat among runners selecting a dominant intrapersonal avoidance goal (*b* = 0.77, *SE* = 0.12, *t* = 6.47, *p* < .01), while the relation was less strong among runners endorsing a dominant intrapersonal approach goal (*b* = 0.31, *SE* = 0.18, *t* = 1.73, *p* = .08).

Regarding the post-race outcomes, regression analyses showed that in Step 1 goal-content predicted performance, with approach-oriented runners running faster when compared to avoidance-oriented runners. When reasons underlying intrapersonal goals were considered in Step 2, autonomous reasons positively predicted need satisfaction, flow, and actual performance. In contrast, controlled reasons related positively to both types of self-talk (i.e., positive and negative). None of the two-way interactions was significant in Step 3. Taken together, the regressions showed the additional predictive validity of autonomous and controlled reasons underlying the pursuit of intrapersonal goals for almost all outcomes.

### Hypothesis 5: Explanatory role of self-talk and need satisfaction

Next, we investigated whether self-talk and need satisfaction in conjunction explain the association between type of intrapersonal goals (i.e., approach-avoidance) along with its underlying reasons and flow. We did not include actual performance because three of the four presumed mediators (i.e., positive and negative self-talk, autonomy and competence need satisfaction) were unrelated to actual performance. Indeed, neither positive self-talk (*r* = .02, *ns*), negative self-talk (*r* = .13, *ns*), or autonomy need satisfaction (*r* = .06, *ns*) were correlated with actual performance among runners endorsing intra-individual goals. Nevertheless, actual performance (expressed in time, so the lower the better) was negatively correlated, as expected, to competence need satisfaction (*r* = –.28, *p* < .01). Also, by constraining the number of included variables in the process model, we kept the ratio of observations to estimated paths at a reasonable level (otherwise the sample would have been shrunk considerably due to listwise deletion).

**Figure 3:**
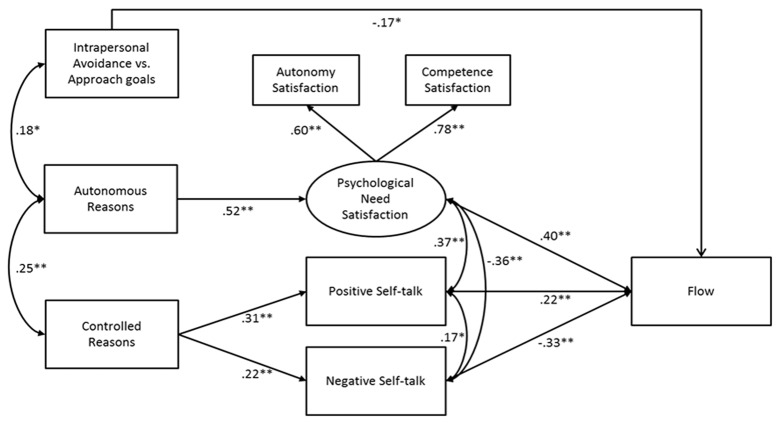
Results of Final Process Model. *Note*. *p < .05; **p < .01.

The process model, shown in Figure 3, yielded the following fit: Satorra-Bentler χ^2^ (18, *N* = 154) = 28.89, *p* = .05, CFI = .953, SRMR = .053, RMSEA = .063 (90%-CI: .002–.103). Consistent with the regression analyses, both positive and negative self-talk were positively predicted by controlled reasons but not by autonomous reasons or type of pursed intrapersonal goal. In turn, positive self-talk was positively and negative self-talk was negatively associated with flow. A follow-up bootstrap analysis of multiple mediators (Preacher & Hayes, 2008) showed a non-significant total indirect effect (*N*= 171; 95%-CI: –.0872; .0401). That is, the two opposing indirect effects through positive (95%-CI: .0329; .1182) and negative self-talk (95%-CI: –.1561; –.0418) evoke two opposing mediational processes, with positive self-talk enhancing and negative self-talk undermining flow.

Further, need satisfaction was positively predicted by autonomous reasons, and in turn positively related to flow. Specifically, the positive indirect effect of autonomous reasons to flow via need satisfaction was significant (β = .21, *z* = 2.92, *p* < .01). Notably, a statistically significant path was found between intrapersonal-avoidance versus intrapersonal-approach goals and flow. This path suggested that intrapersonal-avoidance goals predicted more flow as compared to intrapersonal-approach goals, a finding upon which we return in the discussion.

## Discussion

Drawing upon the intersection between Self-Determination Theory (SDT) and the Achievement Goal Approach (AGA; Vansteenkiste, Lens et al., 2014), we sought to examine whether the type of achievement goals long distance runner set for themselves and the underlying reasons for doing so relate to their race perception, their actual experience of the race, and their performance. Hereby we focused on an understudied type of achievement goals, that is, intrapersonal goals (Elliot et al., 2011), which we deemed to be especially salient in long distance runners. Furthermore, we investigated whether runners’ self-talk and need satisfaction could serve as mediational variables in their goal directed functioning.

### The “What” of Achievement Goals

Consistent with our expectations, the vast majority (i.e., approximately 90%) of the participating long-distance runners preferred a dominant intrapersonal goal over a normative goal. Thus, rather than focusing on outperforming their peers, aiming at their own previous performances seemed to constitute a critical target for these long distance runners. The reduced prevalence of normative goals is consistent with previous studies in the educational (Van Yperen, 2006) and sports domain (Vansteenkiste, Mouratidis, et al., 2014), in which normative goals were also found to be the least prevalent. Of the runners adopting an intrapersonal goal as their primary goal, a larger percentage (i.e., 56.3%) appeared at the starting grid with the goal of improving their last performances. The remaining 43.7% of the runners were focused more dominantly on avoiding performing worse than last time, thus, pursuing an intrapersonal-avoidance goal.

Next, we examined whether runners adopting a different dominant achievement goal would report different pre- and post-race outcomes. Overall, in contrast to what can be expected on the basis of the AGA (e.g., Van Yperen, 2006; Elliot et al., 2011), the differences were fairly minimal. The minimal differences can likely be partly explained by (a) the lack of sufficient power due to the small percentage of runners in the normative goal profiles and (b) the fact that we did not take into account to what extent athletes with a particular dominant goal may also have endorsed, yet to a lesser degree, another type of goal (i.e., a multiple goal perspective).

In spite of these statistical and methodological concerns, the effect that emerged consistently was the association between the valence dimension of achievement goals and runners’ aspired as well as actual running time. That is, individuals adopting an approach goal aspired to a faster time prior to the race and also tended to run faster than those adopting an avoidance goal. In subsequent analyses, thereby controlling for the reasons underlying achievement goals, these relations remained statistically significant. Interestingly, the studied mediators (i.e., self-talk and need satisfaction) could not explain these performance effects. Also, threat appraisals, which have been found to be predicted by avoidance goals in the past (Adie, Duda, & Ntoumanis, 2008), were not related to achievement goal-content in this study. It is possible that this inconsistency is due to the way we assessed achievement goals (i.e., the dominant-goal procedure which involves a categorical rather than a continuous measure). As we cannot provide a definite answer, future research will need to revisit this issue and may consider different mediators that could explain the association between achievement goals and performance. Given that intrapersonal-approach goals have been found to relate positively to energy (Elliot et al., 2011), runners who came to the start with such an achievement goal in mind may have felt more energized. Their elevated energy may lead them to aspire more ambitious and sharper times and to overcome potential barriers during the race, leading them to be more successful than their counterparts with an intrapersonal-avoidance dominant goal. However, these approach oriented runners seemed to experience less flow, which is rather against theory and our expectations. Perhaps as they set a more ambitious running time before the race, this might have caused them to be preoccupied by their target time, which may have led them away from flow experience. This explanation is speculative though as we did not find evidence for a negative correlation between aspired running time and flow experience. Future research should replicate this finding, also because the effect did not appear when the underlying reasons and the mediators were not taken into account.

### The ‘Why’ of intrapersonal goals

Extending previous research on the intersection of the AGA and SDT (e.g., Gaudreau, 2012), the present study sought to examine whether the ‘why’ of achievement goals yielded any unique predictive power when intrapersonal goals were studied. This was indeed the case. A number of findings deserve being highlighted. First, the ‘why’ component proved an additional predictive asset next to the ‘what’ component as all studied outcomes were related to either autonomous or controlled reasons underlying intrapersonal achievement goals. Such findings are consistent with several previous studies on the combination of achievement goals and underlying reasons (e.g., Vansteenkiste, Mouratidis, et al., 2010; Michou et al., 2014; Michou, Matos, Gargurevich, Gumus, & Herrera, this issue).

Second, autonomous motivation was characterized by an overall positive pattern: to the extent runners autonomously regulated their intrapersonal goals, they were more ambitious in the time they were targeting, appraised the race more as a challenge, reported greater need satisfaction and flow during the race, and eventually ran faster. Need satisfaction was found to completely account for the positive contribution of autonomous reasons to flow. Interestingly, self-talk was not predicted by autonomous motivation. Perhaps, autonomously motivated runners get so fully absorbed in the running experience itself that they more easily lose track of time and circumstances. Because of their potential reduced preoccupation with their aspired time, they may be less likely to engage in self-talk, either positive or negative. That is, self-talk may constitute a *corrective* motivational tool to boost one’s own motivation. Such a motivational boost may especially be needed if one finds out that one is running behind schedule and thus may surface as a result of encountered need frustration. Future research could more directly tap into runners’ preoccupations with time and their time checking during the race to examine whether it varies as a function of runners’ ‘what’ and ‘why’ of achievement goals and whether it relates to self-talk and flow.

Third, in contrast to the pronounced positive pattern for autonomous motivation, controlled motivation related to fewer outcomes and, if so, yielded a more ambiguous pattern of relations. That is, in contrast to their autonomous counterparts, runners reporting controlled reasons for pursuing an intrapersonal achievement goal seemed more conflicted towards their goal, as illustrated by the fact that they appraised the race both as a challenge and as a threat. Furthermore, the pressure they experienced may have led them to be more preoccupied with their running time and, as a result, get engaged in both positive or negative self-talk to regulate their goal directed behavior. Noticeable, the pattern of results concerning controlled motivation was not as negative as expected. At least three explanations can be rendered here. First of all, the negative effects of controlled motivation might be more readily pronounced in a team sport like soccer where a bad performance may cost a player’s spot on the team. Because failure under pressure has more immediate ramifications, it may come with a more pronounced cost. Second, we only included a few negative outcome variables. As previous studies (e.g., Haerens et al., 2015; Gillet et al., 2014) pointed out that controlled motivation primarily relates to need frustration rather than to low need satisfaction, investigating more negative outcomes may have yielded more significant contributions of controlled motivation. Furthermore, controlled motivation in running may have fewer implications on short-term outcomes like flow and performance, but might surface over time in the form of dropout (Sarrazin et al., 2002). A third explanation for our findings can be that the effect of controlled reasons may be partly due to the type of achievement goal to which they are tied. Past research shows that controlled reasons underlying ‘suboptimal’ goals (i.e., normative goals) yield strong negative patterns (Vansteenkiste, Mouratidis, et al., 2010), while controlled reasons for ‘more adaptive’ goals (i.e., task goals) do not carry these negative effects (Vansteenkiste, Mouratidis, et al., 2014). Given the small number of runners holding a dominant normative goal in the current study, we cannot draw any firm conclusions. Future research should address this limitation by sampling a greater percentage of normative-oriented athletes.

Two other findings deserve to be highlighted. First, a significant interaction between intrapersonal goals and controlled motivation in the prediction of pre-race threat appraisals emerged, indicating that runners holding an intrapersonal-avoidance goal while standing under pressure were especially vulnerable to perceive the race as threatening. Thus, controlled motivation especially related to threat for those who focused on avoiding to do worse than last time. A similar interactive effect was reported by Gillet et al. (2014) who found autonomous reasons to amplify the positive contribution of normative-approach goals on goal attainment (see also Gaudreau & Braaten, this issue). Yet, except for this one interaction, no other significant interactions emerged, which is in line with other studies in the sports domain (e.g., Vansteenkiste, Mouratidis, et al., 2010).

Second, performance was not related to any of the studied mediators, although it was related to achievement goal content and autonomous reasons. That is, contrary to several other studies (e.g., Blanchfield et al., 2014) and our expectations, positive self-talk was unrelated to performance. Yet, whereas in other studies (e.g., Schüler & Langens, 2007) runners were instructed to consciously use positive self-talk to overcome psychological difficulties during a marathon, we did not manipulate self-talk in the present study. Instead, we assessed self-talk in retrospect, through athletes’ reports, that is, as they felt it had naturally occurred during the race. In other words, runners’ use of self-talk was not necessarily a conscious attempt to regulate their ongoing behavior and goal striving, but may rather have emerged naturally. Further, need satisfaction was unrelated to performance, a finding that deviates from work by Mahoney, Gucciardi, Ntoumanis, and Mallet (2014) who found global need satisfaction during the season to relate to better performance in competitive cross country runners via mental toughness. Mahoney et al. measured need satisfaction as a reflection of the whole season, which created mental toughness in athletes and thus better performance in an important end-of-season race. However, in the current study we assessed race specific need satisfaction. It is likely that this situational satisfaction of needs did not contribute to the mental toughness of our recreational runners and so did not facilitate objective performance.

Interestingly, self-talk and need satisfaction both contributed uniquely to the experience of flow. Whereas self-talk, both positive and negative, served as a rather cognitive explanatory process in the relation between controlled motivation and flow, need satisfaction, as an affective experience, played a mediating role between autonomous motivation and runners’ flow experience. Presumably, autonomous goal pursuit allows for a greater process focus, which is conducive to need satisfaction and a stronger immersion in the activity at hand. In contrast, controlled motivated runners may be more outcome-focused, which may trigger greater cognitive intervention in the form of positive or negative self-talk during the race. Although need satisfaction and both forms of self-talk were meaningfully related, the exact direction of the relation between both could not be addressed in the present study given that they were concurrently assessed. Likely, the relation between both variables is bi-directional. For instance, self-talk could emerge as a function of encountered need frustration, but positive self-talk could also allow one to preserve or even increase one’s experience of competence need satisfaction (see De Muynck, Vansteenkiste, Delrue, Aelterman, Haerens, & Soenens, 2015). Future designs should assess need satisfaction and self-talk on multiple occasions to be able to pinpoint the exact relation between both variables. Furthermore, we recommend future studies to include an assessment of need frustration as well as it may be more strongly related to controlled motivation and give more easily rise to or follow from negative self-talk.

### Limitations

Despite our design in which we included a pre- and post-assessment, we cannot draw any causal conclusion based on the current findings. Future experimental research inducing both particular achievement goals and particular underlying reasons before the race (see Benita et al., 2014) could shed light on this issue. Also, all assessments, except for performance, were subjective. It is advisable to complement at least some of the self-reports with more objective measures. Especially self-talk may not well be captured through self-reports due to retrospective bias (e.g., Zourbanos et al., 2011; Zourbanos et al., 2014) and may be complemented by think-aloud procedures, which require participants to verbalize their inherent self-talk or thought content (e.g., Oliver et al., 2008). However, because of practical implications and possible interference with the race we did not opt for this procedure. Instead, we tried to limit the disadvantage of retrospective bias by assessing self-talk as soon as possible after the race and instructing participants to remember the race vividly before answering the questionnaire.

## Conclusion

The present study was among the first to investigate the recently introduced theory of intrapersonal achievement goals and their underlying reasons in the context of sports. Based on the overall results, we conclude that the ‘why’ of achievement goals yields additional explanatory power to the ‘what’ of achievement goals in relation to runners’ race experiences. Specifically, based on the current findings, we encourage runners to focus on improving their own best time (i.e., to adopt an intrapersonal approach goal) for more volitional (i.e., autonomous) reasons in order to feel challenged before the race, to experience flow during the race and to eventually perform better.

## Competing Interests

The authors declare that they have no competing interests.

## References

[1] Adie J. W., Duda J. L., Ntoumanis N. (2008). Achievement goals, competition appraisals, and the psychological and emotional welfare of sport participants. Journal of Sport & Exercise Psychology.

[2] Benita M., Roth G., Deci E. L. (2014). When are mastery goals more adaptive? It depends on experiences of autonomy support and autonomy. Journal of Educational Psychology.

[3] Blanchfield A. W., Hardy J., De Morree H. M., Staiano W., Marcora S. M. (2014). Talking yourself out of exhaustion: the effects of self-talk on endurance performance. Medicine and Science in Sports and Exercise.

[4] Chen B., Vansteenkiste M., Beyers W., Soenens B., Van Petegem S. (2013). Autonomy in family decision making for Chinese adolescents: disentangling the dual meaning of autonomy. Journal of Cross-Cultural Psychology.

[5] Chen L. H., Wu C. H., Kee Y. H., Lin M. S., Shui S. H. (2009). Fear of failure, 2 × 2 achievement goal and self-handicapping: an examination of the hierarchical model of achievement motivation in physical education. Contemporary Educational Psychology.

[6] Deci E. L., Ryan R. M. (2000). The “what” and “why” of goal pursuits: human needs and the self-determination of behavior. Psychological Inquiry.

[7] De Muynck G-J., Vansteenkiste M., Delrue J., Aelterman N., Haerens L., Soenens B. (2015). The effects of valence and style of feedback provision on need satisfaction, self-talk, and perseverance among tennis players: an experimental study. Manuscript submitted for publication.

[8] Dweck C. S. (1986). Motivational processes affecting learning. American Psychologist.

[9] Elliot A. J., Elliot A. J., Dweck C. S. (2005). A conceptual history of the achievement goal construct. Handbook of competence and motivation.

[10] Elliot A. J., Murayama K., Pekrun R. (2011). A 3 × 2 achievement goal model. Journal of Educational Psychology.

[11] Elliot A. J., Thrash T. M. (2001). Achievement Goals and the Hierarchical Model of Achievement Motivation. Educational Psychology Review.

[12] Gaudreau P. (2012). Goal self-concordance moderates the relationship between achievement goals and indicators of academic adjustment. Learning and Individual Differences.

[13] Gaudreau P., Braaten A. Mastery and performance achievement goals of sport participants and their satisfaction, affect, and perceived goal attainment: Does it matter why goals are pursued?. Psychologica Belgica.

[14] Gillet N., Lafrenière M.-A. K., Vallerand R. J., Huart I., Fouquereau E. (2014). The effects of autonomous and controlled regulation of performance-approach goals on well-being: a process model. The British Journal of Social Psychology / the British Psychological Society.

[15] Haerens L., Aelterman N., Vansteenkiste M., Soenens B., Van Petegem S. (2015). Do perceived autonomy-supportive and controlling teaching relate to physical education students’ motivational experiences through unique pathways? Distinguishing between the bright and dark side of motivation. Psychology of Sport and Exercise.

[16] Hardy J., Hall C. R., Hardy L. (2005). Quantifying athlete self-talk. Journal of Sports Sciences.

[17] Harwoord C., Hardy L., Swain A. (2000). Achievement Goals in Sport: a critique of conceptual and measurement issues. Journal of Sport & Exercise Psychology.

[18] Hatzigeorgiadis A., Zourbanos N., Mpoumpaki S., Theodorakis Y. (2009). Mechanisms underlying the self-talk-performance relationship: the effects of motivational self-talk on self-confidence and anxiety. Psychology of Sport and Exercise.

[19] Jackson S. A., Marsh H. W. (1996). Development and validation of a scale to measure optimal experience: the Flow State Scale. Journal of Sport & Exercise Psychology.

[20] Kawabata M., Mallett C. J. (2011). Flow experience in physical activity: examination of the internal structure of flow from a process-related perspective. Motivation and Emotion.

[21] Kowal J., Fortier M. S. (1999). Motivational determinants of flow: contributions from Self-Determination Theory. Journal of Social Psychology.

[22] Lens W., Vansteenkiste M., Kurt P, Géry d’ Y, International Union of Psychological Science (2006). Motivation: About the “why” and “what for” of human behavior. Psychological concepts: an international historical perspective.

[23] Mahoney J. W., Gucciardi D. F., Ntoumanis N., Mallet C. J. (2014). Mental toughness in sport: motivational antecedents and associations with performance and psychological health. Journal of Sport & Exercise Psychology.

[24] Martin A. J. (2006). Personal bests (PBs): a proposed multidimensional model and empirical analysis. The British Journal of Educational Psychology.

[25] Martin A. J., Liem G. A. D. (2010). Academic personal bests (PBs), engagement, and achievement: a cross-lagged panel analysis. Learning and Individual Differences.

[26] McGregor H. A., Elliot A. J. (2002). Achievement goals as predictors of achievement-relevant processes prior to task engagement. Journal of Educational Psychology.

[27] Michou A., Matos L., Gargurevich R., Gumus B., Herrera D. Building on an enriched hierarchical model of achievement motivation: autonomous and controlling reasons underlying mastery goals and need satisfaction. Psychologica Belgica.

[28] Michou A., Vansteenkiste M., Mouratidis A., Lens W. (2014). Enriching the hierarchical model of achievement motivation: autonomous and controlling reasons underlying achievement goals. British Journal of Educational Psychology.

[29] Morris R. L., Kavussanu M. (2009). The role of approach-avoidance versus task and ego goals in enjoyment and cognitive anxiety in youth sport. International Journal of Sport and Exercise Psychology.

[30] Ng J. Y., Lonsdale C., Hodge K. (2011). The Basic Needs Satisfaction in Sport Scale (BNSSS): instrument development and initial validity evidence. Psychology of Sport and Exercise.

[31] Nicholls A. R., Perry J. L., Calmeiro L. (2014). Precompetitive achievement goals, stress appraisals, emotions, and coping among athletes. Journal of Sport & Exercise Psychology.

[32] Nicholls J. G. (1984). Achievement motivation: conceptions of ability, subjective experience, task choice, and performance. Psychological Review.

[33] Oliver E. J., Markland D., Hardy J., Petherick C. M. (2008). The effects of autonomy-supportive versus controlling environments on self-talk. Motivation and Emotion.

[34] Preacher K. J., Hayes A. F. (2008). Asymptotic and resampling strategies for assessing and comparing indirect effects in multiple mediator models. Behavior Research Methods.

[35] Reeve J., Deci E. L. (1996). Elements of the competitive situation that affect intrinsic motivation. Personality & Social Psychology Bulletin.

[36] Sarrazin P., Vallerand R., Guillet E., Pelletier L., Cury F. (2002). Motivation and dropout in female handballers: a 21-month prospective study. European Journal of Social Psychology.

[37] Scheerder J., Breedveld K., Borgers J. (2015). Running across Europe. The rise and size of one of the largest sport markets.

[38] Schüler J., Langens T. A. (2007). Psychological crisis in a marathon and the buffering effects of self-verbalizations. Journal of Applied Social Psychology.

[39] Senko C., Hulleman C. S., Harackiewicz J. M. (2011). Achievement goal theory at the crossroads: old controversies, current challenges, and new directions. Educational Psychologist.

[40] Van Raalte J. L., Brewer B. W., Lewis B. P., Linder D. E., Wildman G., Kozimor J. (1995). Cork! The effects of positive and negative self-talk on dart throwing performance. Journal of Sport Behavior.

[41] Vansteenkiste M., Lens W., Elliot A. J., Soenens B., Mouratidis A. (2014). Moving the Achievement Goal Approach one step forward: toward a systematic examination of the autonomous and controlled reasons underlying achievement goals. Educational Psychologist.

[42] Vansteenkiste M., Mouratidis A., Lens W. (2010). Detaching reasons from aims: fair play and well-being in soccer as a function of pursuing performance-approach goals for autonomous or controlling reasons. Journal of Sport & Exercise Psychology.

[43] Vansteenkiste M., Mouratidis A., Van Riet T., Lens W. (2014). Examining correlates of game-to-game variation in volleyball players’ achievement goal pursuit and underlying autonomous and controlling reasons. Journal of Sport & Exercise Psychology.

[44] Vansteenkiste M., Smeets S., Soenens B., Lens W., Matos L., Deci E. L. (2010). Autonomous and controlled regulation of performance-approach goals: their relations to perfectionism and educational outcomes. Motivation and Emotion.

[45] Van Yperen N. W. (2006). A novel approach to assessing achievement goals in the context of the 2 x 2 framework: identifying distinct profiles of individuals with different dominant achievement goals. Personality & Social Psychology Bulletin.

[46] Van Yperen N. W., Blaga M., Postmes T. (2014). A meta-analysis of self-reported achievement goals and nonself-report performance across three achievement domains (work, sports, and education). PLoS ONE.

[47] Van Yperen N. W., Elliot A. J., Anseel F. (2009). The influence of mastery-avoidance goals on performance improvement. European Journal of Social Psychology.

[48] Van Yperen N. W., Hamstra M. R. W., Van Der Klauw M. (2011). To win, or not to lose, at any cost: the impact of achievement goals on cheating. British Journal of Management.

[49] Zourbanos N., Hatzigeorgiadis A., Chroni S., Theodorakis Y., Papaioannou A. (2009). Automatic Self-Talk Questionnaire for Sports (ASTQS): development and preliminary validation of a measure identifying the structure of athletes’ self-talk. Sport Psychologist.

[50] Zourbanos N., Hatzigeorgiadis A., Goudas M., Papaioannou A., Chroni S., Theodorakis Y. (2011). The social side of self-talk: relationships between perceptions of support received from the coach and athletes’ self-talk. Psychology of Sport and Exercise.

[51] Zourbanos N., Papaioannou A., Argyropoulou E., Hatzigeorgiadis A. (2014). Achievement goals and self-talk in physical education: the moderating role of perceived competence. Motivation and Emotion.

